# A survey of pharmacists' knowledge, attitudes and barriers in pharmaceutical care concept in Poland

**DOI:** 10.1186/s12909-021-02891-6

**Published:** 2021-08-30

**Authors:** Dorota Kopciuch, Anna Paczkowska, Tomasz Zaprutko, Piotr Ratajczak, Elżbieta Nowakowska, Krzysztof Kus

**Affiliations:** Department of Pharmacoeconomics and Social Pharmacy, Poznan University of Medical Sciences, Rokietnicka 7 St., 60-806 Poznań, Poland

**Keywords:** Safety of medicines, Patients’ pharmacotherapy safety, Pharmacist's behavior, Pharmaceutical care, Pharmacists' knowledge

## Abstract

**Background:**

The major goals of pharmaceutical care (PC) are to improve the patient’s quality of life and ensure safety of pharmacotherapy. Inclusion of a pharmacist in the multidisciplinary team caring for the patient and integration of state-of-the-art pharmaceutical services with medical care and nursing are some of the most important challenges that the health care system in Poland is facing.

**Objectives:**

To evaluate the pharmacists attitudes towards practice in, and knowledge of PC in Poland and to identify the barriers in PC provision.

**Methods:**

The study was designed as a multicenter study, conducted among Polish pharmacists. Random sampling technique was employed to select the study group. Face-to-face questionnaire method was used to interview the pharmacists, upon obtaining their prior verbal consent to participate in the study. The study was conducted between January 2017 and September 2019.

**Results:**

Only 15% of the pharmacists have ever attended a training on PC. 72% believed PC provision was necessary to ensure pharmacotherapy safety. Only 63% of the pharmacists believed that preventing and solving health-related and drug therapy problems for patients were their responsibilities. The main reason for non-provision of PC by the pharmacists was the lack of time for such activities, lack of legal regulations, lack of organizational facilities.

**Conclusion:**

This study indicates that implementation of PC is expected in Poland. Educational programs in this respect are urgently needed. PC provision should be included in the curricula of academic pharmaceutical courses.

**Supplementary Information:**

The online version contains supplementary material available at 10.1186/s12909-021-02891-6.

## Introduction

The concept of pharmaceutical care (PC) has affected pharmacy practice. In many countries, pharmacies are places where individuals may receive health advice and assistance in managing their disease with medications [1]. Usually, pharmacists are the first persons approached by patients with health problems who are frequently unable to contact a physician immediately. Furthermore, mean time of medical consultation in Poland is decreasing, and medical examination of a patient is often followed only by issuing a prescription and providing some evasive information [2]. In this situation, provision of PC seems to be of vital importance.

In 1990, Hepler and Strand [3] published the first useful definition of PC. The major goal of PC is to improve the patient’s quality of life (QOL) [4]. PC provides an opportunity to establish a close cooperation between a pharmacist and a physician to ensure optimal pharmacotherapy conditions for the patient to avoid problems of polypharmacy, and offers the possibility of cost reduction resulting from reimbursement of medications dispensed in pharmacies [5–7]. In Poland, pharmaceutical care has first been defined in the Pharmaceutical Chambers Act [8], updated in 2008 in the amended Pharmaceutical Chambers Act (act amending the Pharmaceutical Chambers Act, 2008, Article 1.2). In spite of the clear definition, the legislator failed to lay down detailed by-laws in the pharmaceutical law system which could make pharmaceutical care a reality in the work of pharmacies; hence, PC was not put into practice either prior to or following development of its definition. The latest Act on the Profession of Pharmacist of 2020 seems to be a ray of hope [9] according to which pharmaceutical care is understood as a health service within the meaning of Article 5.40 of the Act of 27 August 2004 on health care services financed from public funds (Journal of Laws of 2020, item 1398, as amended4), provided by a pharmacist and constituting a documented process, in which the pharmacist, in cooperation with the patient and the doctor treating the patient, and, if necessary, with representatives of other medical professions, ensures the proper course of individual pharmacotherapy.

In the scientific context, pharmaceutical care is the impact of the pharmacist in optimizing pharmacotherapy implemented through a range of pharmaceutical services. These services are mainly aimed at optimizing pharmacotherapy and, among other things, at the search for adverse drug interactions or identification of adverse drug reactions. There is also a notable trend, however, to expand the range of pharmacy services by preventive healthcare and instructing patient on the proper use of drugs, as well as extending their knowledge about their disease and its impact on the patient’s life. In practice, therefore, pharmaceutical care, understood as a broad, holistic approach, is implemented through a number of services/pharmaceutical interventions [10].

In spite of PC’s vital role in optimization and ensuring safety of pharmacotherapy, there is still a limited number of research studies on pharmaceutical care in Poland [11]. Research suggests that patients on the one hand often follow complex pharmacological treatment regimens prescribed by several physicians of different specialties, and, on the other hand, choose self-treatment due to aggressive advertising [12, 13]. Inclusion of a pharmacist in the multidisciplinary team caring for the patient are some of the most important challenges that the health care system in Poland is facing in the context of pharmaceutical care. Although according to many reports the issue of PC in Poland is still open to debate, but there are matters requiring clarification, such as the attitude to the ban on advertising services provided at pharmacies [14].

Currently, both public and hospital pharmacies primarily dispense medicines and ensure the quality of the stored and distributed medicinal products. The research issue was to analyse pharmaceutical care among Polish pharmacists. The research question was: “Do Polish pharmacists have the appropriate knowledge and attitude required to provide pharmaceutical care?”. The study objective was to evaluate the attitudes of pharmacists towards and their knowledge and practice of pharmaceutical care concept in Poland and also to identify the barriers in providing PC in Poland.

## Methods

The study was designed as a multicenter, face-to-face questionnaire study, conducted among Polish pharmacists at community pharmacies. The study was conducted by direct interviews in which the surveyor would read the question aloud to the pharmacist and mark it in the survey, or the pharmacist would enter the answers in the survey and the surveyor was present to explain any unclarities. Cluster random sampling technique was employed to select the study group. The study took place in 3 provinces with the highest number of active pharmacists (the Mazowieckie, Wielkopolskie, and Łódzkie provinces [15]. For every province, 2 cities with the largest number of pharmacies registered in the official database kept by the Chief Pharmaceutical Inspectorate were selected. The interviewers were thoroughly trained before the start of the data collection process. Every interviewer explained the study objectives to every pharmacist. The study was conducted between January 2017 and September 2019.

The questionnaire consisted of 11 questions (Tables 2 and 3). Questions in the questionnaire were based on professional references and then submitted for evaluation by social pharmacy experts. In order to assess the accuracy and comprehensibility of the questionnaire, it was pre-tested [16]

on a sample of 150 pharmacists to whom the purpose of the study had been explained (STATISTICA PL 10.0/ StatSOFT; p <0.05; α= 0.05; p<0.005→H_1_ ; p≥0.05→H_0_). Subsequently, the questions were revised. Results of the pre-test were included in the final study since the pre-test had failed to yield any major modifications of the instrument. The first part of the questionnaire contained questions on the pharmacists’ opinions about safety of pharmacotherapy provided by the healthcare center (Q1). They also referred to the definition of PC (Q2) and attending training/courses in the field of PC provision (Q3). The questionnaire also assessed attitude and knowledge of pharmacists in the field of PC provision (Q4-Q10). The questionnaire pharmacists were also allowed to express their opinion about factors which may discourage pharmacists from providing PC (Q11- Q15).

The study was approved by the Bioethics Committee at Poznan University of Medical Sciences (resolution number: 1056/15). Statistical analysis was performed using STATISTICA PL 10.0 (StatSOFT).

Data were expressed as numbers and percentages. Nominal data were analyzed using the chi-square test of independence. All test were considered significant at *p* <0.05 (α= 0.05; p<0.005→H_1_ ; p≥0.05→H_0_) Significant differences between % of group results were determined by analyzing the Test of Proportions (post-hoc test).

## Results

### Study population

The questionnaires were handed out to 899 pharmacists, of which only 400 expressed their verbal consent to participation in the study. The response rate was 44,49%. The mean age of the pharmacists was 38.05 years. Mean duration of the pharmacists’ practice in a pharmacy (years/duration of practice) was 9.13 years. Most of the pharmacists (72,25%) were Masters of Pharmacy (Table 1).

**Table 1:** Demographic characteristics of pharmacists included in the study evaluating their knowledge, attitude, and practice in terms of pharmaceutical care in Poland (n=400)

Characteristic	
Age [years; mean (SD)]	38.05 (7.76)
Sex [female; N (%)]	165 (41,25)
Years of practice [years; mean (SD)]	9.13 (8.02)
Education level; N (%)	
Masters of Pharmacy	289 (72,25)
Doctors of Pharmacy	111 (27,75)*

* statistically significant difference (p<0.05) vs Masters of Pharmacy

### Knowledge

Most of the pharmacists were familiar with the definition of pharmaceutical care (PC) (76%) (Table 2). Knowledge of the above definition was correlated both with age (*p*<0.05) and education level (*p*<0.05), i.e. younger and better educated pharmacists had a better knowledge of the PC definition (Table 2). Interestingly, the concept and main assumptions of the PC were known mostly by people with longer work experience and age (Table 2).

**Table 2:** Assessment of pharmacists’ knowledge, attitude and practice towards pharmaceutical care (PC) concept and policy (n=400)

Questions	Response N (%)	Age (%)				Education level (%)		Years of practice (%)		
		<30	31-40	41-50	>50	Masters of Pharmacy	Doctors of Pharmacy	<10	11-20	>20
**Knowledge**										
What is the definition of PC? Correct Answer	304 (76%)	47	26	14	13*	39	61	25**	32	43
		p < 0.05				p < 0.05		p < 0.05		
Have you ever had a course/attended a workshop on PC? Answer: Yes	60 (15%)	82^	18	0	0	55	45	69	23	8^a^
		p < 0.05				NS		p < 0.05		
**Attitudes**										
Do you believe that the healthcare system guarantees pharmacotherapy safety? Answer: Yes	172 (43%)	15	12^b^	35	38	36	64	30	21^c^	49
		p < 0.05				p < 0.05		p < 0.05		
Do you believe that the primary aim of pharmaceutical care is to improve and maintain the patient’s quality of life? Answer: Yes	172 (43%)	12^d^	29	37	22	45	55	15^e^	44	41
		p < 0.05				NS		p < 0.05		
Do you believe PC provision is necessary to ensure pharmacotherapy safety? Answer: Yes	328 (82%)	33	54	10^f^	3^g^	46	54	61	32^h^	7^i^
		p < 0.05				p < 0.05		p < 0.05		
Are you willing to provide pharmaceutical counselling? Answer: Yes	180 (45%)	49	26	18	7^j^	43	57	23^k^	47	30
		p < 0.05				p < 0.05		p < 0.05		
Do you believe preventing and solving health-related and drug therapy problems to be your responsibilities? Answer: Yes	272 (68%)	33	40	14	13^l^	53	47	35	38	27^m^
		p < 0.05				NS		p < 0.05		
Do you believe that the future success of the pharmacy will depend on provision of professional services in addition to dispensing? Answer: Yes	300 (75%)	35	39	20	6^n^	69	31	41	47	12***
		p < 0.05				p < 0.05		p < 0.05		
**Practice**										
Do your patients frequently ask you for advice on pharmacotherapy? Answer: Yes	276 (69%)	27	37	23	13^^	45	55	40	47	13^^^
		p < 0.05				p < 0.05		p < 0.05		
Do you contact a physician if you suspect a drug interaction? Answer: Yes	128 (32%)	34	66	0^#^	0^&^	62	38	43	57	0^z^
		p < 0.05				p < 0.05		p < 0.05		

* statistically significant difference (p<0.05) vs <30 y.o.a. and 31-40 y.o.a.; ** statistically significant difference (p<0.05) vs >20 years; ^statistically significant difference (p<0.05) vs 31-40, 41-50 and >50 y.o.a; ^a^ statistically significant difference (p<0.05) vs <10 years and 11-20 years; ^b^ statistically significant difference (p<0.05) vs 41-50 and >50 y.o.a; ^c^statistically significant difference (p<0.05) vs >20 years; ^d^statistically significant difference (p<0.05) vs 31-40, 41-50 and >50 y.o.a; ^e^ statistically significant difference (p<0.05) vs 11-20 and >20 years; ^f^statistically significant difference (p<0.05) vs <30 y.o.a., 31-40 and >50 y.o.a; ^g^statistically significant difference (p<0.05) vs <30 y.o.a., 31-40 and 41-50 y.o.a; ^h^ statistically significant difference (p<0.05) vs <10 and >20 years; ^i^statistically significant difference (p<0.05) vs <10 years and 11-20 years; ^j^statistically significant difference (p<0.05) vs <30, 31-40 and 41-50 y.o.a; ^k^significant difference (p<0.05) vs 11-20 and >20 years; ^l^statistically significant difference (p<0.05) vs <30, 31-40 and 41-50 y.o.a; ^m^statistically significant difference (p<0.05) vs <10 years and 11-20 years; ^n^statistically significant difference (p<0.05) vs <30, 31-40 and 41-50 y.o.a; *** statistically significant difference (p<0.05) vs <10 years and 11-20 years; ^^statistically significant difference (p<0.05) vs <30, 31-40 and 41-50 y.o.a; ^^^statistically significant difference (p<0.05) vs <10 years and 11-20 years; ^#,&^statistically significant difference (p<0.05) vs <30, 31-40 y.o a.; ^z^statistically significant difference (p<0.05) vs <10 years and 11-20 years

Only 15% (N=60) of the pharmacists have ever attended a training on PC; this correlated both with age (*p*<0.05) professional experience (*p*<0.05) (Table 2).

### Attitudes and practice

Forty-three percent (43%) of pharmacists believed that the healthcare system guaranteed safety of pharmacotherapy (Table 2). This opinion was correlated with the pharmacists’ age (older) (*p*<0.05), education (*p*<0.05) and duration of practice (*p*<0.05); namely, older pharmacists with a lower level of education and longer work experience were more frequently convinced about safety of the pharmacotherapy guaranteed by the system.

Most pharmacists (82%) believed PC provision was necessary to ensure pharmacotherapy safety. This belief was correlated with age (*p*<0.05), education level (*p*<0.05), and duration of professional practice (*p*<0.05), i.e. younger and better educated pharmacists, with a shorter professional practice, had a stronger belief that PC provision was necessary (Table 2). Only 45% were willing to provide pharmaceutical counselling, and the younger and better educated ones were more willing to advise the patients on safety of their pharmacotherapy. Only 68% of the pharmacists believed that preventing and resolving health-related and drug therapy problems for patients were their responsibilities, and 75% claimed that future success of their pharmacy would depend on provision of professional services in addition to dispensing. This belief was correlated with the pharmacists’ age and duration of professional practice (*p*<0.05), i.e. younger pharmacists, with a shorter professional practice, were more positively pre-disposed to the issues addressed in the questions. The need for pharmaceutical counselling provision was reported by 69% of the pharmacists who claimed that their patients frequently asked them for advice on their pharmacotherapy (Table 2). Only thirty-two percent (32%) of the pharmacists claimed they consulted a physician if they suspected a drug interaction (Table 2).

### Barriers

The main reason for non-provision of PC by the pharmacists was the lack of time for such activities (87%), lack of legal regulations (87%), lack of organizational facilities (87%), insufficient knowledge of PC (79%), and no need for PC provision (37%) (Table 3). These results were mostly correlated with sociodemographic data (*p*<0.05) (Table 3).

**Table 3:** Factors which may discourage pharmacists from delivering pharmaceutical care (n= 400)

Questions	Response N(%)	Age (%)				Education level (%)		Years of practice (%)		
		<30	31-40	41-50	>50	Masters of Pharmacy	Doctors of Pharmacy	<10	11-20	>20
Lack of time to delivering PC	348 (87%)	26	39	26	9*	58	42	33	35	32
		p<0.05				p<0.05		NS		
Lack of legal regulations	348 (87%)	23	37	28	12	56	44	33	35	32
		NS				p<0.05		NS		
Lack of organizational facilities (e.g. a dedicated room) for PC provision	348 (87%)	23	31	26	20	43	57	40**	28	32
		NS				p<0.05		p<0.05		
Level of knowledge makes it difficult to delivering PC	316 (79%)	50	17	5^	32	41	49	53	15^^	32
		p<0.05				p<0.05		p<0.05		
Don't feel the need to delivering PC	148 (37%)	40	32	23	5^#^	47	53	20	23	57^&^
		p<0.05				NS		p<0.05		

*statistically significant difference (p<0.05) vs <30 y.o.a, 31-40 y.o.a. and 41-50 y.o.a; **statistically significant difference (p<0.05) vs 11-20 and >20 years; ^^^statistically significant difference (p<0.05) vs <30 y.o.a, 31-40 y.o.a. and >50 y.o.a; ^^statistically significant difference (p<0.05) vs <10 and >20 years; ^#^statistically significant difference (p<0.05) vs <30 y.o.a, 31-40 y.o.a. and 41-50 y.o.a; ^&^statistically significant difference (p<0.05) vs <10 and 11-20 years

## Discussion

The results of the present study primarily demonstrate that the majority of pharmacists in Poland see the need to provide PC and the need to regulate the legal and organizational issues of PC.

The need for PC implementation in Poland has been previously noted by other Polish researchers [10, 11, 17, 18], and by the National Section of Pharmaceutical Care of the Polish Pharmaceutical Society which presented their own proposal of state-of-the-art pharmaceutical care development in Poland in 2016 [19] addressing the areas for improvement and issues limiting provision of PC in Poland in detail.

Our study has shown that in spite of the lack of any legal regulations of PC practice in Poland, pharmacists are positively pre-disposed towards the PC concept. These attitudes are similar to those reported in other countries such as Thailand and New Zealand [20, 21]. The results of our study have shown that 57% of pharmacists know the definition of PC, 43% of pharmacists understand its concept, and 78% believe that future success of pharmacy depends on provision of professional services in addition to dispensing medicines. Similar observations were made in a study conducted in New Zealand [20] which showed, among other things, that over 60% of the pharmacists surveyed had a correct understanding of PC. Approximately the same percentage in that study felt that the future of pharmacy would depend on provision of services other than dispensing medicines.

Furthermore, the results of our study emphasize that pharmacists with longer job experience have a better understanding of PC concept statements compared to less experienced pharmacists. This was similar to the results from Saudi Arabia and Nigeria [22, 23] Both our study and the study conducted in Qatar [24] have shown that pharmacists with more experience have less positive attitudes towards PC concept. A probable explanation for this study finding is that pharmacists who have more experience may be hesitant to provide PC given all the inherent obstacles that may hinder PC implementation that they have seen in their years of practice. These pharmacists are more prone to acknowledge the difficulties entailed with starting a new service.

Preparation of pharmacists for PC provision was another aspect addressed by our study. It was shown that although Polish pharmacists are positively inclined towards PC, few of them are well prepared to carry out this type of task. Only 15% of pharmacists declared that they had ever attended a PC course/training. Similar observations were made in the Wajid et al. study [25] in which pharmacists reported lack of adequate training in the field of PC as one of the main barriers to its implementation, thus emphasizing the role of universities as places where they should acquire such knowledge. 83% of pharmacists participating in the study in Saudi Arabia received their highest pharmaceutical degree at universities where schools of pharmacy follow traditional science-based undergraduate pharmaceutical curricula that are not designed to produce pharmacists with sufficient knowledge and skills to provide optimal PC.

Introduction of PC-related subjects to the basic curriculum of pharmacy students is necessary, especially that the patients themselves emphasize such a necessity. In our study, as many as 66% of pharmacists declared that patients frequently asked them for pharmacotherapy-related advice, proving the patients’ positive attitude towards PC.

Data available in the literature also indicate that patients’ experiences result in their positive attitude towards PC [26] and make them expect an increasingly wide range of the new service [27]. The impact of PC implementation on pharmacotherapy safety has been confirmed by numerous research studies carried out worldwide, proving PC’s health benefits for the patients, such as improved therapy safety and efficacy parameters, specifically the adherence (to recommendations) or reduced self-treatment ratios [21–24].It is of particular importance since many prescribed medications are not taken according to the doctor’s recommendations which contributes to an increase in the number of patients admitted to hospitals and, thus, increased expenditure on healthcare [28]. An appropriate intervention of a pharmacist, however, helps prevent medication problems, resulting in the desired effects of the pharmacotherapy prescribed [29–31].

In spite of PC’s tangible positive importance for ensuring pharmacotherapy safety for the patients, PC provision encounters a number of barriers. In Poland, lack of legal regulations is the main barrier, as declared by as many as 87% of pharmacists. Other barriers equally frequently reported by pharmacists include lack of time (87%), lack of organizational facilities (87%), level of knowledge (76%), and feeling there is no need for PC provision (37%). This was consistent with results of other studies. For example, lack of time was the main barrier to non-provision of PC in Australia, Argentina, China, New Zealand, Portugal, and Thailand where pharmacists also claimed that dispensing takes too much time and, thus, they are reluctant to be involved in other activities [21, 32–35]. Further barriers, such as the lack of organizational facilities (e.g. lack of privacy and lack of space), were also a problem among pharmacists from Qatar and Spain [36] where pharmacists claimed that incorporation of a private or semi-private counseling area and of a patient waiting area at their pharmacies would facilitate the pharmacist-patient encounter, enhancing the privacy of conversations, and improving the counselling atmosphere [37]. Another barrier reported in our study – level of knowledge on PC – was a major limitation for PC provision in such countries as Thailand or Qatar. Results of the study conducted in Qatar [37] showed that 64% of pharmacists reported inadequate training in PC. In a study conducted in Thailand [21], the pharmacists also reported that they lacked therapeutic knowledge and clinical problem-solving skills and had little confidence in providing pharmaceutical care. This attitude towards PC was also reported in other studies [38, 39].

Limited interactions between medical professionals was another, equally important barrier reported in our study. Our study has shown that as few as 32% of respondents claimed they consulted a physician when they suspected a drug interaction, which corroborated with reports of other study authors who also emphasise this as an issue requiring extra attention [40, 41].

The PC concept does not undermine the responsibilities of other healthcare providers. Physicians and nurses have well recognized and important roles in the PC process. Strong cooperative relationships between pharmacists and other healthcare providers are necessary in order to design an appropriate care plan for the patient’s drug-related problems and to optimize their therapeutic outcomes. The popularization of interprofessional collaboration should be launched in concert with the undergraduate training and subsequently continued in the postgraduate education. The future should bring enhanced efforts of all relevant stakeholders in the dissemination of the concept of interprofessional collaboration, predominantly from the practical point of view [10]. To effectively implement the cognitive services, legal-related issues should be resolved, particularly those related to medical information [42]. To do this, pharmacists need to have access to the patients’ medical histories.

Finally, the issue of legal regulations and remuneration for PC provision are major factors contributing to the process of successful implementation of pharmaceutical care in Poland. Both the government and health insurance companies should understand that PC services are both clinically and economically effective, and that an appropriate reimbursement system should be put in place for PC services rendered by pharmacists. Financial aspects should be resolved prior to full implementation of new services. Furthermore, pharmaceutical care is dedicated to chronically ill and elderly populations. Hence, the services should be reimbursed from public sources. Otherwise, the roll-out of these services will be rather marginal [10].

According to the Supreme Pharmaceutical Council, the Act on the Profession of a Pharmacist, the draft of which was submitted for social consultation this July, would give a go-ahead to PC provision in Poland. Works are currently under way at the Ministry of Health on pharmaceutical care piloting, and feature meetings of the Team for pilot implementation of pharmaceutical care whose job is to develop and suggest solutions for testing the pharmaceutical care system at community pharmacies. Work is also pending on the Minister for Health’s Regulation on pharmaceutical care pilot program which will lay down the rules of keeping records of pharmaceutical care and the method of accounting for the payments received from the budget of the National Health Fund. Hopefully, these works will bring us closer to practical implementation of PC in Poland.

### Limitations

The study population was relatively small (n=522) and may be difficult to be generalized, and the responses of the pharmacists may also have been biased. The study was also limited by the probability of recall bias due to different professional experience, seniority, and age of the pharmacists. Moreover, the lack of validity and reliability measures for the questionnaire should also be considered as another limitations of the study.

## Conclusion

This study indicates that formal implementation of PC is expected by community of pharmacists’ in Poland.

Results of this study also show, however, that Polish pharmacists still have poor knowledge of the PC concept. Educational programs in this respect are urgently needed. PC provision should be included in the curricula of academic pharmaceutical courses. Lack of legal regulations and, hence, lack of remuneration and organizational conditions for PC provision in pharmacists’ everyday work is also a huge limitation.

## Supplementary Information

**Additional file 1.**

## Data Availability

not applicable

## Abbreviations

PC: Pharmaceutical care
QOL: Quality of life
