# Supplementary material for: A survey of pharmacists' knowledge, attitudes and barriers in pharmaceutical care concept in Poland
Source: BMC Med Educ. 2021 Aug 30;21:458. doi: 10.1186/s12909-021-02891-6 (PMC8404347; doi:10.1186/s12909-021-02891-6)
Supplement: Supplementary file 1 — Additional file 1. [file 12909_2021_2891_MOESM1_ESM.docx]

**Questionnaire**

**1. What is the definition of PC?**

a. It is the direct provision of medication-related care. It is provided by a pharmacist in which the pharmacist ensures the proper course of individual pharmacotherapy.

b. It is the direct, responsible provision of medication-related care for the purpose of achieving definite outcomes that improve a patient's quality of life. It is documented process, in which the pharmacist, in cooperation with the patient and the doctor treating the patient, and, if necessary, with representatives of other medical professions, ensures the proper course of individual pharmacotherapy

c. Pharmaceutical care is the provision of medication-related care for the purpose of achieving definite outcomes that improve a patient's quality of life. It is the health service provided by a pharmacist in cooperation with physicians in which the pharmacist ensures the proper course of individual pharmacotherapy and proper patients’ quality-of-life.

**2. Have you ever had a course/attended a workshop on PC?**

a. Yes

b. No

**3. Do you believe that the healthcare system guarantees pharmacotherapy safety?**

a. Yes

b. No

**4. Do you believe that the primary aim of pharmaceutical care is to improve and maintain the patient’s quality of life?**

a. Yes

b. No

**5. Do you believe PC provision is necessary to ensure pharmacotherapy safety?**

a. Yes

b. No

**6. Are you willing to provide pharmaceutical counselling?**

a. Yes

b. No

**7. Do you believe preventing and solving health-related and drug therapy problems to be your responsibilities?**

a. Yes

b. No

**8. Do you believe that the future success of the pharmacy will depend on provision of professional services in addition to dispensing?**

a. Yes

b. No

**9. Do your patients frequently ask you for advice on pharmacotherapy?**

a. Yes

b. No

**10. Do you contact a physician if you suspect a drug interaction?**

a. Yes

b. No

**11. Please, indicate the factors which may discourage pharmacists from delivering pharmaceutical care.**

a. Lack of time to delivering PC

b. Lack of legal regulations

c. Lack of organizational facilities (e.g. a dedicated room) for PC provision

d. Level of knowledge makes it difficult to delivering PC

e. Don't feel the need to delivering PC
